# Midostaurin in daily clinical practice of patients with advanced systemic mastocytosis

**DOI:** 10.1111/bjh.70065

**Published:** 2025-08-08

**Authors:** Johannes Lübke, Nicole Naumann, Timo Brand, Laurenz Steiner, Roland Repp, Georgia Metzgeroth, Alice Fabarius, Wolf‐Karsten Hofmann, Deepti H. Radia, Andreas Reiter, Juliana Schwaab

**Affiliations:** ^1^ Department of Hematology and Oncology University Hospital Mannheim, Heidelberg University Mannheim Germany; ^2^ Medical Department 2 City Hospital Kiel Kiel Germany; ^3^ Haematology Department Guy's and St Thomas' NHS Foundation Trust London UK

**Keywords:** advanced systemic mastocytosis, dosing profiles, efficacy, KIT inhibitor, midostaurin, multikinase inhibitor, safety

## Abstract

Midostaurin, a multikinase/KIT inhibitor, is approved for the treatment of advanced systemic mastocytosis (AdvSM). We evaluated dosing regimens, safety, efficacy and overall survival (OS) of 79 patients from the ‘German Registry on Disorders of Eosinophils and Mast Cells’. Midostaurin was initiated at 200 and ≤150 mg daily in 63/79 (80%) and 16/79 (20%) and continued at month 12 in 44/63 (70%) and 10/16 (63%) patients respectively. Over a cumulative observation period of 146 patient‐years, 96 adverse events (AE) led to dose adjustment, most commonly nausea/emesis (*n* = 22, 23%) and neutropenia (*n* = 8, 8%). Responses were achieved in 60% (modified Valent criteria), 29% (IWG‐MRT‐ECNM, International Working Group‐Myeloproliferative Neoplasms Research and Treatment & ECNM criteria) and 13% (pure pathological response criteria) of patients. Within the first 12 months, achievement of a response was dose independent except for modified Valent criteria. The response duration (modified Valent/IWG‐MRT‐ECNM criteria) correlated with improved OS (*p* < 0.001). Midostaurin was ultimately stopped in patients due to lack of response/progression, death and AEs in 28 (39%), 5 (6%) and 13 (17%) patients respectively. Following midostaurin discontinuation, 7/41 (17%) patients experienced a discontinuation syndrome, which was effectively prevented in subsequent patients through dose tapering and corticosteroid bridging. In conclusion, midostaurin demonstrated a favourable safety profile and yielded durable responses in AdvSM patients across dosing regimens.

## Graphical abstract

Durable responses based on modified Valent, IWG‐MRT‐ECNM and PPR criteria were predominantly dose‐independent, making dose adjustments due to better tolerability more practicable in daily routine. Gradual tapering of midostaurin, prophylactic treatment with antihistamines and corticosteroids and timely initiation of subsequent therapy can prevent a severe discontinuation syndrome. AdvSM, advanced systemic mastocytosis; AE(s), adverse event(s); AHN, associated haematological neoplasm; ASM, aggressive systemic mastocytosis; CRP, C‐reactive protein; DOR, duration of response; INR, international normalized ratio; IWG‐MRT‐ECNM, International Working Group‐Myeloproliferative Neoplasms Research and Treatment & ECNM; MCL, mast cell leukaemia; ORR, overall response rate; Pyoderma gangren., Pyoderma gangrenosum.

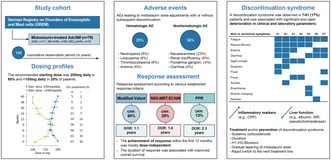

## INTRODUCTION

Systemic mastocytosis (SM) is a rare myeloid neoplasm characterized by multifocal accumulation of neoplastic mast cells (MC) predominantly in the bone marrow (BM) and visceral organs. According to the 2022 revised World Health Organization (WHO‐5) and International Consensus Classification (ICC), advanced systemic mastocytosis (AdvSM) comprises aggressive SM (ASM), SM with an associated haematological (WHO‐HAEM5)/myeloid (ICC) neoplasm (SM‐AHN/SM‐AMN) and mast cell leukaemia (MCL).^1^, ^2^, ^3^, ^4^

Significant advances have been made in elucidating the genetic landscape of SM.^5^, ^6^, ^7^, ^8^, ^9^, ^10^, ^11^, ^12^, ^13^, ^14^, ^15^, ^16^ The canonical driver mutation *KIT* D816V is present in ≥80% of AdvSM patients and arises in early haematopoietic stem and progenitor cells, leading to a multilineage non‐MC restricted involvement.^17^ Moreover, 70%–80% of AdvSM patients harbour additional somatic mutations with *TET2*, *SRSF2*, *ASXL1* and *RUNX1* being the most frequently affected genes.^5^, ^10^, ^11^ The combination of selected high‐risk mutations (e.g. *SRSF2*, *ASXL1*, *RUNX1*) with other prognostically relevant clinical characteristics has led to the development of several risk scoring systems for overall (OS) and progression‐free survival (PFS).^18^, ^19^, ^20^, ^21^

A single‐arm, phase 2, nonrandomized registrational study enrolling 89 evaluable patients^22^ has established the multikinase/KIT‐inhibitor midostaurin for treatment of patients with AdvSM. In a propensity score‐weighted analysis aimed to address confounders, midostaurin showed superior efficacy over the purine analogue cladribine, which has been widely used off‐label for nearly two decades.^21^

Given the clinical uncertainty surrounding the impact of different midostaurin dosing profiles on safety, treatment response and survival outcomes, a cohort of 79 AdvSM patients enrolled within the ‘German Registry on Disorders of Eosinophils and Mast Cells’ (GREM) was analysed.

## PATIENTS AND METHODS

### Study population

Between 2009 and 2021, 125 AdvSM patients were treated with midostaurin at the Department of Hematology and Oncology, University Hospital Mannheim, Germany.^3^, ^4^ For a thorough analysis of response in these patients, integrated analysis of clinical data and sequential BM biopsies was performed in 79 patients (Table S1). All BM biopsies were reviewed by reference pathologists of the European Competence Network on Mastocytosis (ECNM). Diagnosis of AdvSM was made between 1997 and 2021 and classified according to the WHO‐HAEM5 classification.^1^ All patients would also have met the diagnostic criteria of the International Consensus Classification (ICC).^2^ The cohort included patients with ASM (*n* = 11, 14%), SM‐AHN (*n* = 50, 63%) and MCL ± AHN (*n* = 18, 23%). The most common associated haematological neoplasm (AHN) (63/79, 80%) diagnoses were myelodsysplastic neoplasm (MDS)/myeloproliferative neoplasm (MPN) (24/63, 30%), chronic myelomonocytic leukaemia (CMML) (21/63, 33%), chronic eosinophilic leukaemia (CEL) (6/63, 10%) and MDS (5/63, 8%). The median age at treatment initiation was 66 years (range 25–87) and 48 (61%) patients were male. The median time to treatment initiation was 0.8 years (range 0–19.4 years). The cumulative observation period on midostaurin was 146 patient‐years with a median treatment duration of 1.0 year (range 0.1–11.4). Progression into secondary MCL and secondary AML occurred in 9/79 (11%) and 7/79 (9%) patients respectively. At time of data cut‐off, 31/79 patients (39%) remained on‐treatment. Patients were enrolled within the ‘German Registry on Eosinophils and Mast Cells (GREM)’.

The study design adhered to the tenets of the Declaration of Helsinki and was approved by the Institutional Review Board of the Medical Faculty Mannheim, Heidelberg University (Heidelberg, Germany). All patients gave written informed consent.

### Assessment of response and adverse events

Response assessment was performed according to the (i) modified Valent criteria,^22^, ^23^ (ii) the modified International Working Group‐Myeloproliferative Neoplasms Research and Treatment & ECNM (IWG‐MRT‐ECNM) criteria^24^, ^25^ and (iii) the pure pathological response (PPR) criteria.^25^ PPR criteria were recently established on patients treated with avapritinib within the EXPLORER (phase I) and PATHFINDER (phase II) studies (Tables S2–S4). In contrast to the post hoc analysis on midostaurin by food and drug administration (FDA), the response category ‘clinical improvement’ was included within the overall response rate (ORR) of this retrospective study, which is in line with the response analysis by European Medicines Agency (EMA).

All BM biopsies were evaluated by reference pathologists of the ECNM.^26^ BM biopsies were available at diagnosis or treatment initiation and at ≥1 subsequent time point during midostaurin therapy. The *KIT* D816V expressed allele burden (EAB) was measured by quantitative allele‐specific polymerase chain reaction on RNA level as previously described.^6^ Only adverse events (AE) leading to dose modification were collected. Grading of AEs was performed using the Common Terminology Criteria for Adverse Events (CTCAE), Version 5.0.^27^

### Statistical analyses

Continuous variables were analysed for statistical differences using the Student's *t*‐test. If the values were not normally distributed, a Wilcoxon rank‐sum test was employed. For categorical variables, Fisher's exact test was used. We retrospectively analysed the event‐free survival (EFS, time from treatment initiation to the date of disease progression according to modified Valent criteria, IWG‐MRT‐ECNM criteria or PPR criteria/last follow‐up [if alive]) by using the Kaplan–Meier method with log‐rank test for group comparisons/visualizations. The proportional hazards assumption was tested by the correlation of scaled Schoenfeld residuals with time. *p* values of <0.05 (two‐sided) were considered statistically significant. Data management and statistical analyses were performed using R version 4.3.1 (R Foundation for Statistical Computing, Vienna, Austria), SPSS version 29.0.1 (IBM Corporation, Armonk, NY) and GraphPad Prism version 10.1.1 software (GraphPad Software Inc., San Diego, CA, USA).

## RESULTS

### Starting dose and dose modification

**FIGURE 1:**
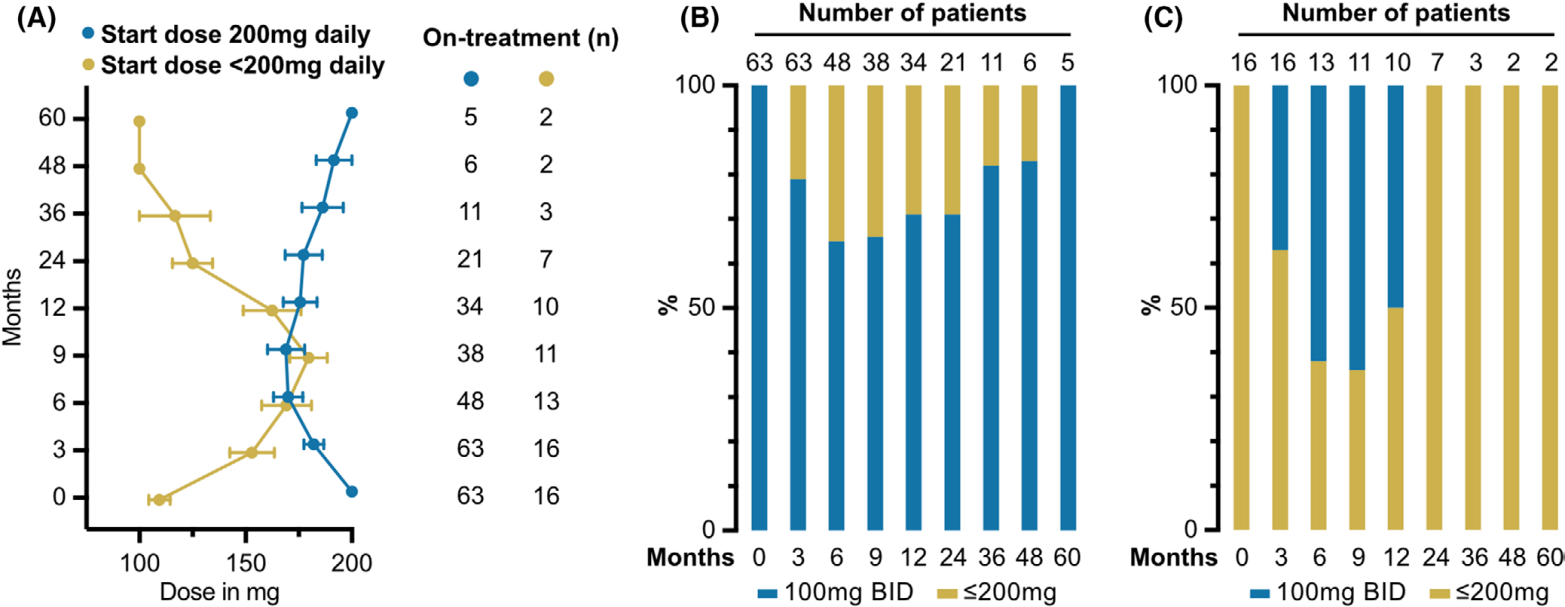
Dosing profile at initiation and over disease course. (A) Comparison of dose profiles of patients with a starting dose of 200 mg (including patients with a prespecified dose escalation) and patients with a starting dose of <200 mg. (B) Dose profile over time in patients with a starting dose of 200 mg. (C) Dose profile over time in patients with a starting dose of <200 mg. *N*, number.

The starting dose was 200 mg daily (100 mg BID), 150 mg daily (variable) or 100 mg daily (50 mg BID) in 63/79 (80%), 2/79 (2%) and 14/79 (18%) patients respectively. In the 200 mg daily cohort, the starting dose was maintained at month 3, 6, 9, 12, 24 and 36 in 50/63 (79%), 30/48 (63%), 25/38 (66%), 23/34 (68%), 15/22 (68%) and 9/9 (100%) patients respectively. During follow‐up, 25/63 (40%) patients were reduced to ≤150 mg daily. Due to a better drug tolerability in the evening, the 150 mg dose was usually split into 50 mg‐0‐100 mg. In the combined cohorts of 150 mg daily and 100 mg daily starting dose (16/79, 20%), the dose was maintained at month 3, 6, 9, 12, 24 and 36 in 10/16 (63%), 5/13 (38%), 4/11 (36%), 5/10 (50%), 7/7 (100%) and 3/3 (100%) patients. The dose could be increased to 200 mg daily in 9/16 (56%) patients (Figure 1).

### Reasons for dose modification

In 146 patient‐years, 96 AEs led to dose modifications in 38/79 (48%) patients. AEs were observed across all AdvSM subtypes and dose regimens. Haematological and non‐haematological toxicities accounted for 24 (25%) and 36 (38%) of AEs, with neutropenia (8/96, 8%) and nausea/emesis (22/96, 23%; female, 17/22, 77%) being the most common. The most frequent AE grade 3/4 was neutropenia (4/96, 4%). The retrospective character of this analysis did not allow adequate grading of non‐haematological AEs. Prophylactic and supportive treatment with variable doses of 5HT3‐antagonists was administered in 64 (81%) patients. Nausea/emesis was the sole reason for stopping midostaurin in only 2 (4%) patients. Gastrointestinal biopsies obtained prior to midostaurin were available in 8/22 (36%) patients and did not reveal evidence of increased MC infiltration as a potential cause of nausea/emesis (Table 1).

**TABLE 1:** Adverse events leading to midostaurin dose adjustment/stopp.

	Reasons for dose adjustment/discontinuation^a^	
	Any grade	Grade ≥3
Overall, *n* (%)	96 (100)	12 (13)
Hematologic AEs, *n* (%)	24 (25)	12 (13)
Neutropenia	8 (8.3)	4 (4.2)
Leucopenia	6 (6.3)	4 (4.2)
Thrombocytopenia	6 (6.3)	3 (3.1)
Anaemia	4 (4.2)	1 (1.0)
Non‐haematological AEs, *n* (%)	36 (38)	
Nausea/emesis	22 (23)	—
Renal insufficiency	6 (6.3)	—
Pyoderma gangrenosum	3 (3.8)	—
Diarrhoea	3 (3.8)	—
Cardiac decompensation	2 (2.1)	—
Hepatic toxicity	1 (1.0)	—
Periorbital oedema	1 (1.0)	—
Other reasons (not related to AE)^b^	36	

*Note*: Grade ≥3 was not evaluable for non‐haematological AEs.

Abbreviation: AE, adverse event.

^a^ One patient may have multiple dose adjustments/discontinuations and can therefore contribute to multiple AEs.

^b^ Other reasons included dose adjustment/discontinuation according to response/resistance, initiation of other treatment options.

### A yet unreported adverse event: Pyoderma gangrenosum

Three cases of pyoderma gangrenosum were diagnosed 2, 3 and 12 months after the start of midostaurin 200 mg daily with CI as best responses per IWG‐MRT‐ECNM criteria. All three patients presented with rapidly enlarging, painful ulcers with a necrotic centre surrounded by erythema. In patients #1 and #2, the ulcers were located on the lower leg, while in patient #3, it developed at the stoma site following complete resection of stage 1 colon cancer (Figure S1). Histology of the biopsies showed no evidence of MC infiltration. Midostaurin was stopped and all patients were treated with oral corticosteroids (starting dose 1 mg/kg, followed by tapering). Following the resolution of the pyoderma gangrenosum to a partially atrophic scar within 2 months, patient #1 was switched to cladribine, followed by allogeneic haematopoietic cell transplantation. The patient remains under close follow‐up with no signs of disease progression and is currently (+5 years post alloHCT) receiving ruxolitinib for chronic graft‐versus‐host disease of the oral mucosa. Patients #2 and #3 were both switched to avapritinib (#2: dose 200 mg daily, duration 6 months, no response; #3: dose 200 mg daily, duration 2 months, no response). At the follow‐up visit after 3 months of avapritinib treatment in patient #2, the ulcer showed marked clinical improvement. Central necrotic debris had diminished, peripheral erythema had faded, and the violaceous border was less pronounced. Although a shallow central ulceration persisted, no new lesions were observed. In patient #3, one and a half months later, the follow‐up visit revealed near‐complete epithelialization of the lesion, with only a small residual central ulcer. The surrounding skin showed post‐inflammatory hyperpigmentation, with no signs of ongoing inflammation.

### Comparison of response criteria

**FIGURE 2:**
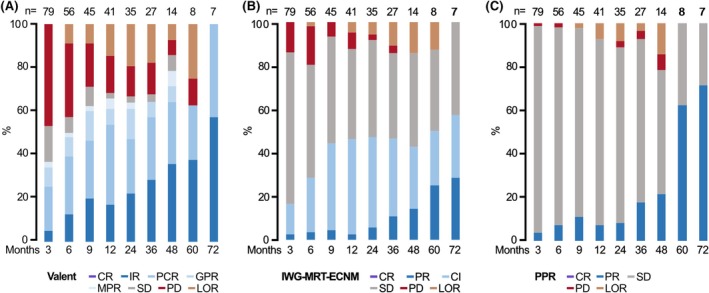
Response assessment according to different response criteria. Response assessment according to (A) modified Valent criteria, (B) International Working Group‐Myeloproliferative Neoplasms Research and Treatment & European Competence Network on Mastocytosis criteria (IWG‐MRT‐ECNM criteria), and (C) Pure pathological response criteria (PPR criteria). According to modified Valent, IWG‐MRT‐ECNM or PPR criteria, the overall response rate was 60% (ASM, *n* = 8, 73%, SM‐AHN, *n* = 27, 54%, MCL ± AHN, *n* = 12, 67%), 29% (ASM, *n* = 4, 36%, SM‐AHN, *n* = 13, 26%, MCL ± AHN, *n* = 6, 33%) and 13% (ASM, *n* = 1, 9%, SM‐AHN, *n* = 6, 12%, MCL ± AHN, *n* = 3, 17%) with a median time to best response of 0.2, 0.5 and 0.8 years respectively. CI, clinical improvement; CR, complete response; GPR, good partial response; IR, incomplete response; LOR, loss of response; MPR, minor partial response; *n*, number; PCR, pure clinical response; PD, progressive disease; PR, partial response; SD, stable disease.

According to modified Valent, IWG‐MRT‐ECNM or PPR criteria, the ORR was 60%, 29% and 13% with a median time to best response of 0.2, 0.5 and 0.8 years respectively (Figure 2). No patient achieved a complete response. The median DOR was 1.1, 1.4 and 2.3 years respectively. Only for the PPR criteria, achievement of a response was associated with improved OS (*p* = 0.032), whereas DOR correlated with improved OS and deepening HRs over time according to modified Valent criteria and IWG‐MRT‐ECNM criteria (Table 2). In comparison to modified Valent criteria, the IWG‐ECNM‐MRT criteria upstaged 27 (34%) patients into a superior response category and downstaged 17 (22%) patients into an inferior response category (Figure S2). Median EFS was 0.3, 0.8 and 1.2 years respectively.

**TABLE 2:** Response assessment for midostaurin.

Response criteria assessment within overall duration of treatment	Univariable analysis		
	*n*	HR (95% CI)	*p*
Modified Valent criteria (response)^a^: yes vs. no	79	0.676 (0.390–1.170)	0.162
Duration of response (months)			
≥6		**0.215 (0.101–0.458)**	**<0.001**
≥12		**0.183 (0.079–0.426)**	**<0.001**
≥18		**0.242 (0.190–0.538)**	**<0.001**
IWG‐MRT‐ECNM response criteria (response)^b^: yes vs. no	79	0.852 (0.476–1.524)	0.589
Duration of response (months)			
≥6		0.667 (0.148–3.012)	0.598
≥12		**0.245 (0.087–0.690)**	**0.008**
≥18		**0.238 (0.074–0.763)**	**0.016**
Pure pathological response criteria (response)^c^: yes vs. no	79	**0.355 (0.137–0.915)**	**0.032**
Duration of response (months)			
≥6		0.280 (0.046–1.706)	0.167
≥12		0.166 (0.018–1.570)	0.117
≥18		0.166 (0.018–1.570)	0.117

*Note*: Statistically significant values are shown in bold.

Abbreviations: CI, confidence interval; HR, hazard ratio; IWG‐MRT‐ECNM, International Working Group‐Myeloproliferative Neoplasms Research and Treatment & European Competence Network on Mastocytosis; *n*, evaluable number of patients.

^a^ Gotlib et al.^22^

^b^ Gotlib et al.^24^

^c^ Shomali et al.^25^

### Impact of dosing profile on response

**FIGURE 3:**
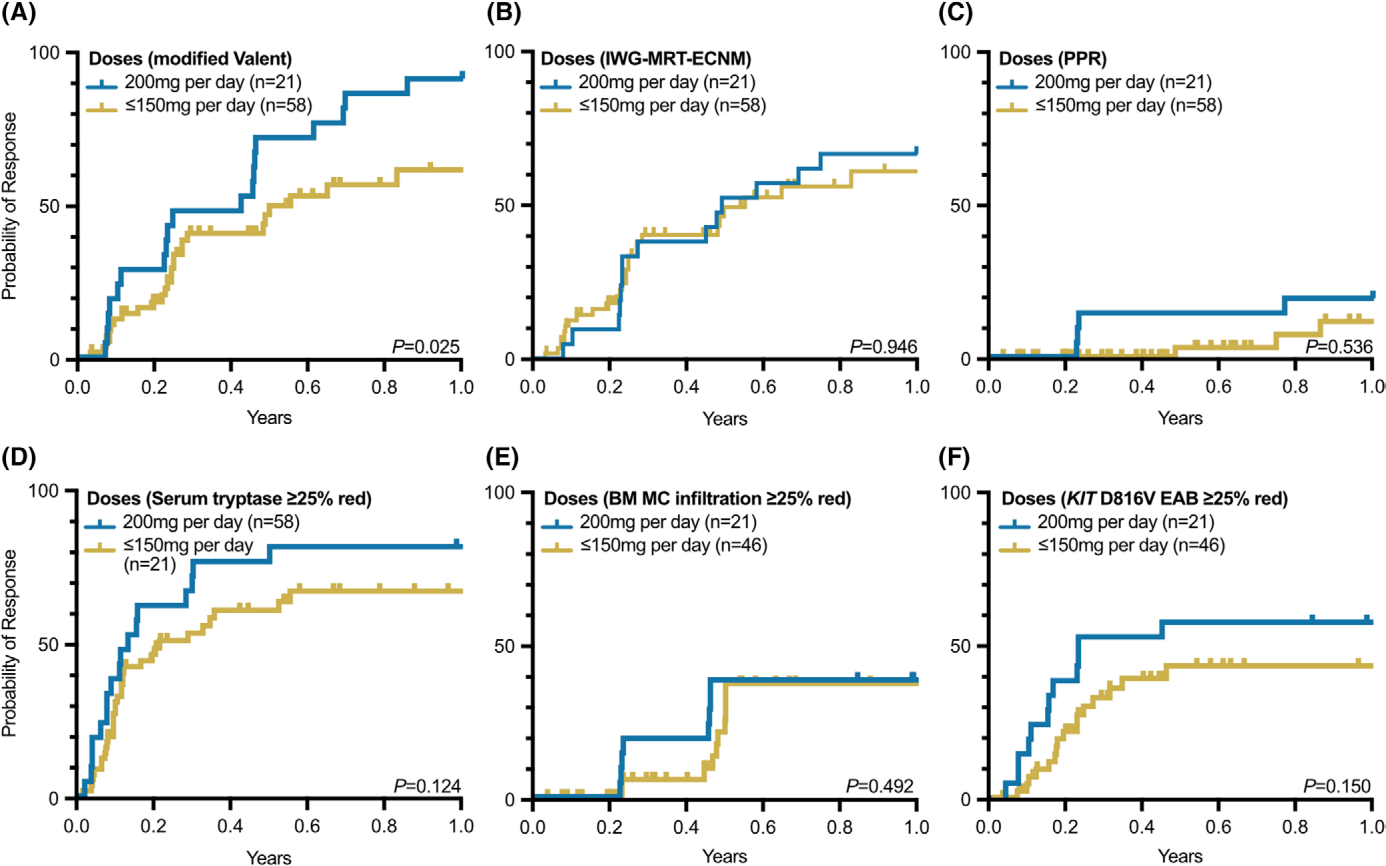
Achievement of response. A response based on serum tryptase, bone marrow (BM) mast cell (MC) infiltration, and KIT D816V expressed allele burden (EAB) levels was considered valid when a reduction of at least 25% was attained. IWG‐MRT‐ECNM, International Working Group‐Myeloproliferative Neoplasms Research and Treatment & European Competence Network on Mastocytosis criteria; PPR, Pure Pathologic Response criteria.

The achievement of a response of serum tryptase (*p* = 0.124), BM MC infiltration (*p* = 0.492) and *KIT* D816V EAB levels (*p* = 0.150) was dose‐independent (Figure 3). Within the first 12 months, the achievement of a response was dose‐independent (200 mg daily vs. any reduced dose) according to IWG‐MRT‐ECNM (*p* = 0.946) and PPR criteria (*p* = 0.536) while it was dose dependent according to modified Valent criteria (*p* = 0.025).

### Midostaurin discontinuation syndrome

**FIGURE 4:**
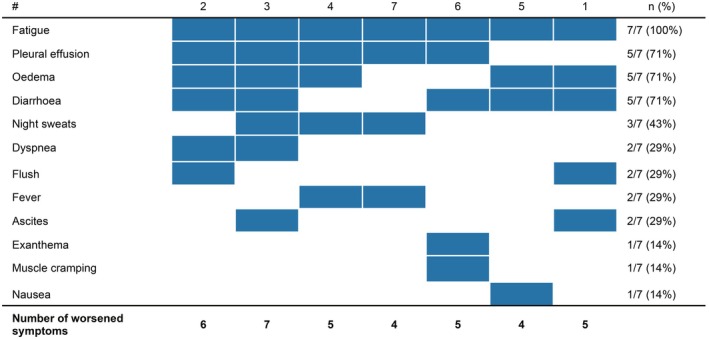
Midostaurin discontinuation syndrome. Symptom profile at time of midostaurin discontinuation syndrome. *N*, number.

Among 41 evaluable patients, seven patients (17%; MCL, *n* = 3; SM‐MDS/MPN, *n* = 3; MCL‐MDS/MPN, *n* = 1) experienced significant and rapid deterioration in clinical and laboratory parameters after discontinuation of midostaurin due to progressive disease (PD)/loss of response (LOR) by IWG‐MRT‐ECNM criteria (Table 3, Figure 4). The median time to symptom onset and subsequent consultation following discontinuation was 14 days (range 7–35). The most commonly reported symptoms were deterioration of fatigue (7/7, 100%), peripheral oedema (5/7, 71%), pleural effusion (5/7, 71%) and diarrhoea (5/7, 71%). Hospitalization was required in 5/7 (71%) patients.

**TABLE 3:** Patient characteristics prior and at time of midostaurin discontinuation syndrome.

#	1		2		3		4		5		6		7	
Age^a^, sex	60, f		77, f		69, m		65, m		83, f		59, m		71, f	
	Pre	Post	Pre	Post	Pre	Post	Pre	Post	Pre	Post	Pre	Post	Pre	Post
Blood counts														
WBC, ×10^9^/L	6.0	10.7	7.7	30.6	3.1	3.8	1.5	2.1	5.9	11.3	7.1	10.6	7.5	4.6
Haemoglobin (g/dL)	8.0	6.5	11.3	11.7	11.6	10.1	8.7	8.3	10.3	10.8	12.0	12.2	10.7	7.3
Platelets, ×10^9^/L	20	10	59	54	114	175	92	51	88	89	235	193	61	36
Serum chemistry														
Tryptase (μg/L)	1194	981	643	578	450	535	384	550	200	276	150	250	288	—
Vitamin B12 (U/L)	502	430	285	368	305	—	—	—	664	854	245	151	—	—
AP (U/L)	479	452	155	113	128	212	347	228	122	375	92	191	65	68
Albumin (g/L)	27.6	12.7	43.2	32.9	41.9	34.7	36.0	27.9	37.4	31.0	41.0	35.3	42.8	31.0
Cholinesterase (U/L)	4934	2644	13 666	7036	10 570	6276	8723	6056	6981	4718	8930	4212	5395	3510
INR	1.11	1.25	1.18	1.96	1.27	1.69	1.22	1.67	1.08	1.35	1.12	1.54	1.13	1.12
CRP (mg/dL)	10	19	9	102	8	15	17	76	9	24	17	21	45	74
Treatment														
Last midostaurin dose (mg/day)	50		150		150		100		200		200		100	
Time to consultation (days)	7		13		35		14		14		14		21	
Hospitalization	Yes		Yes		Yes		No		Yes		No		Yes	
Subsequent treatment line	Ava^b^		Ava^b^		Ava^b^		Ava^b^		Clad^b^		Ava^b^		Clad	
TtNT (weeks)	4.4		4.4		5.2		4.0		2.8		4.0		0.4	

Abbreviations: AP, alkaline phosphatase; Ava, Avapritinib; Clad, Cladribine; d, day(s); CRP, C‐reactive protein; INR, internalized ratio; TtNT, time to next treatment line; WBC, white blood cells.

^**a**^ Age at symptom onset.

^b^ Bridging with prednisolone 20 mg/day was initiated, with gradual tapering based on clinical symptoms.

The development of a discontinuation syndrome was seen independently of the last midostaurin dose. Laboratory evaluations before and after discontinuation revealed significant changes in liver function (median albumin 41 vs. 31 mg/dL, *p* = 0.027; cholinesterase 8.7 vs. 4.7 U/mL, *p* = 0.024; INR 1.13 vs. 1.45, *p* = 0.489) and inflammatory markers (median white blood cells 6.0 vs. 10.6 × 10^9^/L, *p* = 0.223; C‐reactive protein: 10 vs. 24 mg/dL, *p* = 0.065). Treatment of midostaurin discontinuation syndrome included the initiation of systemic corticosteroids (e.g. prednisolone 20–50 mg daily), diuretics (torasemide/furosemide, dose depending on clinical symptom burden), and, where not already established, H1‐/H2‐blockers. No further cases of a midostaurin discontinuation syndrome were observed after implementing gradual tapering of the midostaurin dose, the prophylactic use of systemic corticosteroids (20–50 mg daily) and a more rapid switch to the next treatment line.

## DISCUSSION

In patients with AdvSM, the recommended starting dose of midostaurin is 200 mg daily (100 mg BID). For the prevention of frequently occurring nausea (as described by patients: ‘starting one hour after intake and lasting for one hour’) but rarely occurring emesis, the use of prophylactic antiemetics, such as 5HT3 receptor antagonists, is recommended based on physician's and patient's preference. Patients should be advised to take midostaurin with food which enhances absorption by approximately 22% (or 59% with a high‐fat meal) and decreases peak concentration (Cmax) by 20% (27% with a high‐fat meal).^28^ From our personal experience, we also recommend taking the evening dose shortly before bedtime, as the nausea does not usually wake patients up. In our cohort, the recommended starting dose of 200 mg daily was applied in 80% of patients and could be maintained in the majority of patients. Particularly in elderly or comorbid patients, we initiated treatment at 50% or 75% starting dose. A substantial proportion of these patients were able to increase at least the evening dose to 100 mg, while this was less frequently possible with the morning dose.

The improvement of serum tryptase, BM MC burden, cytopenias, gastrointestinal C‐findings and *KIT* D816V variant allele burden is variably included within different response assessment criteria of AdvSM.^29^ The global registrational trial identified an ORR of 60% (45% major responses) by modified Valent criteria.^22^ In post hoc analyses by EMA/FDA, an ORR of 28%/17% was reported according to IWG‐MRT‐ECNM criteria because FDA decided to exclude the category clinical improvement from the ORR.^30^ Real‐world ORR according to Valent criteria was reported in the vicinity of 42%–71%. In our real‐world cohort, we observed an ORR of 60% and 29% according to modified Valent and IWG‐MRT‐ECNM criteria, respectively. The ORR according to PPR criteria was only 13%, reflecting the very strict threshold values,^25^ but the achievement of a response was associated with improved OS. According to modified Valent criteria, a higher dose was associated with a higher ORR, while the achievement of an ORR within the first 12 months was largely dose independent according to IWG‐MRT‐ECNM and PPR criteria. These findings suggest that a higher dose may be beneficial in some cases but is not necessarily needed for the achievement of a response. However, a dose–response relationship cannot be ruled out over the longer course of midostaurin treatment.

Differentiating treatment‐related AEs from disease‐related symptoms is inherently complex, as they may frequently coincide with both lack of response or secondary resistance. The concurrent presence and the frequently observed heterogeneous response of SM and AHN make decisions even more complex. AEs were common, with a significant proportion of patients experiencing at least one event leading to (temporarily) dose modification or discontinuation. For reasons of reliability, we only included AEs that resulted in subsequent dose modification/discontinuation. The most frequent haematological and nonhaematological AEs were neutropenia and nausea respectively. A rare but severe AE, pyoderma gangrenosum, was observed in three patients. In a retrospective analysis of 116 patients with a pyoderma gangrenosum, 13/116 (11%) were found to have an underlying haematological neoplasm, though no cases of a MPN were identified. The specific treatment approaches were not described.^31^ A systematic literature review identified 15 case reports of pyoderma gangrenosum as a probable and reversible adverse reaction to TKIs, including two cases involving imatinib.^32^, ^33^, ^34^, ^35^

In *BCR::ABL1* positive chronic myeloid leukaemia, the ABL1‐inhibitor imatinib is frequently stopped after the achievement of a deep molecular remission with the aim of a durable treatment‐free remission. Approximately 20% of patients reported the occurrence of a musculoskeletal pain, which is predominantly treated by anti‐inflammatory drugs.^36^ In classical MPN such as myelofibrosis, discontinuation of the JAK1/JAK2 inhibitor ruxolitinib is challenging because symptom control may persist despite objective indicators of disease progression.^37^, ^38^, ^39^, ^40^, ^41^, ^42^ A systematic database analysis reported a ‘ruxolitinib discontinuation syndrome (RDS)’ in 14% of patients (34/251).^43^, ^44^, ^45^, ^46^, ^47^ RDS included a variable pattern of worsening splenomegaly, cytopenias and, in severe cases, a life‐threatening septic shock‐like syndrome. Among patients with MPNs, discontinuing ruxolitinib during COVID‐19 was associated with a higher risk of mortality compared to those who continued treatment.^48^

Midostaurin exerts significant effects on MC activation. Specifically, it has been demonstrated to inhibit immunoglobulin E (IgE)‐dependent activation and mediator release in human MCs and basophils.^49^, ^50^, ^51^ This suppression of mediator release contributes to the alleviation of MC‐related symptoms in patients undergoing treatment. Upon abrupt discontinuation of midostaurin, patients may experience a revivification of MC activity due to the removal of this inhibitory effect. In seven patients of our cohort, a dose‐independent clinical deterioration occurred within median of 14 days of midostaurin discontinuation, which was variably characterized by fatigue, pleural effusion, oedemas and diarrhoea, in addition to worsening of liver function and CRP in the absence of any apparent infection. A subtle elevation of CRP was already present at the time of discontinuation in all seven patients, highlighting the chronic inflammatory nature of the disease.^52^ The role of corticosteroids in this context is noteworthy. Corticosteroids are known to suppress MC mediator release, providing a therapeutic option to mitigate the effects of MC reactivation.^53^, ^54^, ^55^ In our cohort, the implementation of corticosteroids, alongside antihistamines and diuretics, proved effective in managing the discontinuation syndrome. In summary, a gradual tapering of midostaurin, rather than the abrupt discontinuation, combined with the prophylactic use of antihistamines and short‐term corticosteroids, proved highly effective in preventing discontinuation symptoms in subsequent patients.

In conclusion, durable responses based on modified Valent, IWG‐MRT‐ECNM and PPR criteria were predominantly dose‐independent making dose adjustments due to better tolerability more practicable in daily routine. Gradual tapering of midostaurin, prophylactic treatment with antihistamines and corticosteroids and timely initiation of subsequent therapy can prevent a severe discontinuation syndrome.

## AUTHOR CONTRIBUTIONS

JL, NN and AR and JS have assessed and verified the data. JL, AR and JS contributed to the concept and design and involved in the interpretation of data. JL, NN, TB, LS, RR, AR and JS were involved in the acquisition of data. JL and JS contributed to the statistical analysis. All authors were involved in manuscript writing. All authors contributed to the critical revision of the manuscript and for important intellectual content, read and approved the final manuscript and are accountable for all aspects of the work.

## FUNDING INFORMATION

JL and NN are supported by the ‘Wilhelm Sander‐Stiftung’ (grant no. 2023.120.1).

## CONFLICT OF INTEREST STATEMENT

JL received honoraria from Blueprint Medicines. DR has been a clinical advisory board/study steering group member (EXPLORER/PATHFINDER) for Blueprint Medicines Corporation; a study steering committee member for Cogent Biosciences; and involved in educational events and advisory boards for Novartis. Author fees for Medscape cases and Fast Facts: Systemic Mastocytosis. AR has received research funding, served on advisory boards, received honoraria and funding to cover travel expenses from AbbVie, AOP, Blueprint Medicines Corporation, BMS, GSK, Incyte and Novartis. JS reports honoraria from Blueprint Medicines, Cogent, GSK, Novartis and Astra Zeneca. Also reports research funding from GSK, Blueprint and Cogent.

## ETHICS STATEMENT

The study design adhered to the tenets of the Declaration of Helsinki and was approved by the institutional review board of the Medical Faculty Mannheim, Heidelberg University (Heidelberg, Germany).

## PATIENT CONSENT STATEMENT

All patients gave written informed consent.

## Supporting information


Data S1.

## Data Availability

The datasets used and/or analysed during the current study are available from the corresponding author on reasonable request.
