# Supplementary material for: Midostaurin in daily clinical practice of patients with advanced systemic mastocytosis
Source: Br J Haematol. 2025 Aug 8;207(4):1388–96. doi: 10.1111/bjh.70065 (PMC12512096; doi:10.1111/bjh.70065)
Supplement: Supplementary file 1 — Data S1. [file BJH-207-1388-s001.docx]

**Supplementary information**

**Impact of Midostaurin Dosing Profiles on Safety and Response in Patients with**

**Advanced Systemic Mastocytosis**

Lübke J, Naumann N, Brand T, Steiner L, Repp R, Metzgeroth G, Fabarius A, Hofmann WK, Reiter A, Schwaab J

**Table of contents**

**Supplementary Figures**

**Figure S1:** Pyoderma gangrenosum ………….………...…………...…….……………………………………………..2

**Figure S2:** Integrated response assessment………...…………...…….……………………………………………..3

**Supplementary Tables**

**Table S1:** Comparison of baseline characteristics: full vs. analyzed midostaurin cohort……………4

**Table S2:** Modified Valent response criteria………...…………...…………………………………………………..5

**Table S3:** IWG-MRT-ECNM response criteria.…………………………………………………………..……...…….6

**Table S4:** PPR response criteria.……………………………………………………………..…………………..………….8

**Figure S1: Pyoderma gangrenosum**

|  |  |  |
| --- | --- | --- |
| 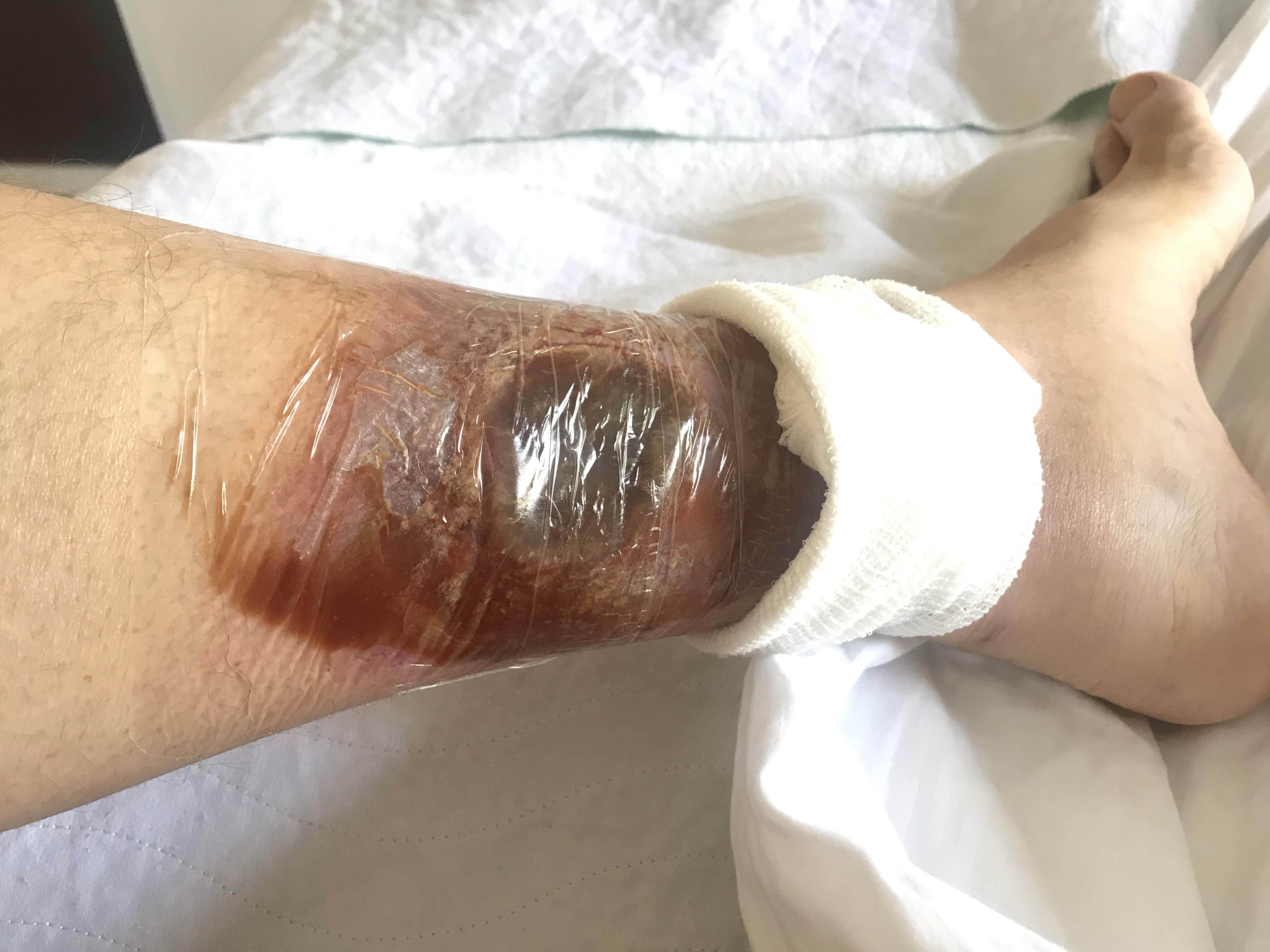  A | 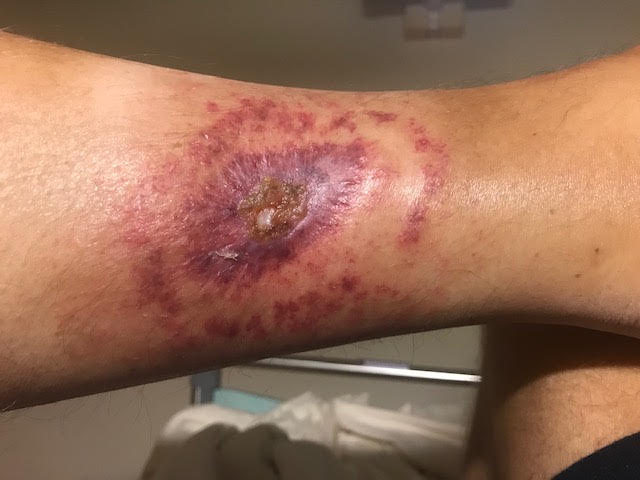  B | 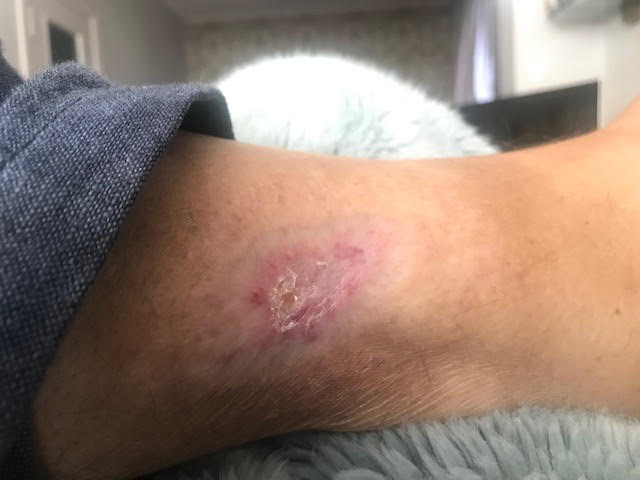  C |
|  |  |  |
|  |  |  |
| 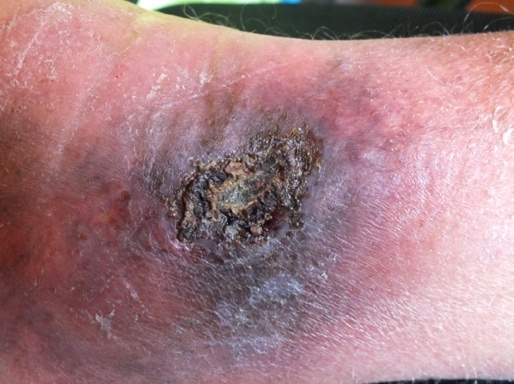  D | 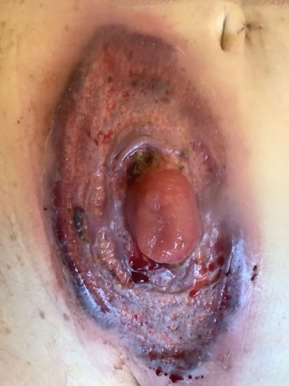  E |  |

Figure panels A.-C. illustrate the gradual improvement of pyoderma gangrenosum in patient #1. Panels D. and E. correspond to patient #2 and patient #3, respectively. No follow-up images were available.

**Figure S2: Integrated response assessment.**

Responses were re-categorized based on comparisons between **A.** the modified Valent criteria and ECNM-IWG-MRT criteria, **B.** the modified Valent criteria and PPR criteria and **C.** the ECNM-IWG-MRT criteria and PPR criteria.

Abbreviations: IWG-MRT-ECNM, International Working Group-Myeloproliferative Neoplasms Research and Treatment & European Competence Network on Mastocytosis criteria; n, number, PD, progressive disease; PPR, Pure Pathologic Response criteria; R, response, SD, stable disease.

| **Table S1: Comparison of baseline characteristics: full vs. analyzed midostaurin cohort** | | | | | |
| --- | --- | --- | --- | --- | --- |
|  | |  | ***Full cohort*** |  | ***Analyzed cohort*** |
| Number of patients, *n* (%) | |  | N=125 |  | n=79 |
| Age in years at treatment initiation; median (range) | |  | 67 (22-87) |  | 66 (25-87) |
| Male, *n* (%) | |  | 83 (66) |  | 48 (61) |
|  | |  |  |  |  |
| **WHO-HAEM5 diagnosis** | |  |  |  |  |
|  | ASM, *n* (%) |  | 19 (15) |  | 11 (14) |
|  | SM-AHN, *n* (%) |  | 82 (66) |  | 50 (63) |
|  | MCL±AHN, *n* (%) |  | 24 (19) |  | 18 (23) |
|  |  |  |  |  |  |
| **AHN diagnosis** | |  |  |  |  |
|  | CMML *n* (%) |  | 33 (26) |  | 21 (27) |
|  | MDS/MPN *n* (%) |  | 30 (24) |  | 24 (30) |
|  | MDS *n* (%) |  | 9 (7) |  | 5 (6) |
|  | CEL *n* (%) |  | 13 (10) |  | 6 (8) |
|  | Others *n* (%) |  | 10 (8) |  | 7 (9) |
|  |  |  |  |  |  |
| **C-findings** | |  |  |  |  |
|  | Hemoglobin <10 g/dL, *n* (%) |  | 53 (42) |  | 35 (44) |
|  | Platelet count <100 x10^9^/L, *n* (%) |  | 54 (43) |  | 34 (43) |
|  | Absolut neutrophil count <1 x10^9^/L, *n* (%) |  | 5 (4) |  | 3 (4) |
|  | Alkaline phosphatase >150 U/L, *n* (%) |  | 74 (59) |  | 52 (66) |
|  | Albumin level <34 g/L, *n* (%) |  | 45 (36) |  | 34 (43) |
|  |  |  |  |  |  |
| **Other relevant findings** | |  |  |  |  |
|  | Leukocytes ≥16 x10^9^/L, *n* (%) |  | 28 (22) |  | 16 (20) |
|  | Monocytes ≥1 x10^9^/L, *n* (%) |  | 40 (32) |  | 29 (37) |
|  | Eosinophils ≥1.5 x10^9^/L, *n* (%) |  | 24 (11) |  | 12 (15) |
|  | Bone marrow mast cell burden ≥50%, *n* (%) |  | 48 (38) |  | 34 (43) |
|  | Serum tryptase levels ≥125 ng/ml, *n* (%) |  | 73 (58) |  | 29 (37) |
|  | *KIT* D816V expressed allele burden ≥10%, *n* (%) |  | 78 (62) |  | 48 (61) |
|  |  |  |  |  |  |
| Abbreviations: ASM, aggressive systemic mastocytosis; BM, bone marrow; MCL, mast cell leukemia; MCL-AHN, mast cell leukemia with an associated hematologic neoplasm; SM-AHN, systemic mastocytosis with an associated hematological neoplasm. | | | | | |

| **Table S2: Modified Valent response criteria.^21^** | | | | | |
| --- | --- | --- | --- | --- | --- |
| **Response** | **C-findings ^a^** | **Subcategory** | **Mast cell infiltration in organ** | **Serum tryptase level** | **Organomegaly** |
| Major response | ≥1 C-finding resolved | Complete remission | None | <20 μg/L | None |
|  |  | Incomplete remission | >50% | >50% | >50% |
|  |  | Pure clinical response | No significant change | ≤50-0% | No significant change |
|  |  | Unspecified | Cannot be subtyped **^b^** | | |
| Partial response | ≥1 C-finding improved by >50% | Good partial response **^c^** |  | | |
|  | ≥1 C-finding improved by >20-≤50% | Minor partial response |  |  |  |
| No response | C-findings show constant range | Stable disease |  |  |  |
|  | ≥1 C-finding worsened by >20% | Progressive disease |  |  |  |
| Only measurable C-findings were eligible: transfusion-independent and -dependent anemia and thrombocytopenia; neutropenia; liver function abnormalities (increased alanine aminotransferase, aspartate aminotransferase, and/or total bilirubin); hypoalbuminemia; medically documented weight loss ≥10% in the 6 months prior to the study. Ascites and bone lesions were not permitted as sole C-findings because they were not considered quantifiable.  **^a^** For major response, partial response, or stable disease, no C-findings could show progression.  **^b^** Subtype could not be determined due to lack of bone marrow involvement and/or liver/spleen measurement and/or serum tryptase levels.  **^c^** Minimum increases required for hematologic response: hemoglobin, 1.5 g/dL; absolute neutrophil count, 0.2×109/L; platelets, 20×109/L. | | | | | |

| **Table S3: (Modified) IWG-MRT-ECNM response criteria.^23^** | | | |
| --- | --- | --- | --- |
|  | **IWG-MRT-ECNM definition** | **IWG-MRT-ECNM response criteria** | **mIWG-MRT-ECNM modifications** |
| **Non-hematologic organ damage** | | | |
| Ascites or pleural effusions | Symptomatic ascites or pleural effusion requiring medical intervention such as: Use of diuretics (grade 2) or  ≥2 therapeutic paracenteses or  thoracenteses (grade 3) at least 28 days apart over 12 weeks before the start of treatment with one procedure performed 6 weeks before the start of treatment | Complete resolution of symptomatic ascites or pleural effusion (including trace/minimal on radiographic imaging) and no longer in need of diuretics for ≥12 weeks and  No longer in need of diuretics for ≥12 weeks or  No therapeutic paracenteses or  thoracentesis for ≥12 weeks | Same as IWG-MRT-ECNM |
| Liver function abnormalities | ≥Grade 2 abnormalities in direct bilirubin (>1.5 × ULN), AST (>3.0 × ULN), ALT (>3.0 × ULN), or ALP (>2.5 × ULN) in the presence of: Ascites and/or Clinically relevant portal hypertension, and/or Liver MC infiltration that is biopsy-proven or No other identified cause of abnormal liver function | Reversion of ≥1 LFTs to normal range for ≥12 weeks | Same as IWG-MRT-ECNM |
| Hypoalbuminemia | ≥Grade 2 hypoalbuminemia  (<3.0 g/dL) | Reversion of albumin to normal range for ≥12 weeks | Same as IWG-MRT-ECNM |
| Marked symptomatic  splenomegaly | A spleen that is palpable >5 cm below the left costal margin and patient endorses symptoms of discomfort and/or early satiety | ≥50% reduction in palpable splenomegaly (or ≥35% reduction in spleen volume based on 3D MRI or CT scan) and no endorsement of discomfort and/or early satiety for ≥12 weeks | Definition: Symptomatic or non-symptomatic splenomegaly palpable ≥5 cm below left costal margin. Response criteria: ≥35% reduction in spleen volume based on 3D MRI or CT scan for ≥12 weeks |
| Weight loss | N/A | N/A | Definition: Medically documented >10% weight loss in last 24 weeks ( ± 12 weeks) Response criteria: Reversion of >50% of weight loss in the 24 weeks preceding treatment |
| **Hematologic organ damage** | | | |
| Neutropenia | ≥Grade 3 ANC (<1.0 × 10^9^/L) | ≥100% increase and an absolute increase ≥0.5 × 10^9^/L for ≥12 weeks | Same as IWG-MRT-ECNM with allowance of CRh **^a^** |
| Anemia (transfusion-independent) | ≥Grade 2 Hb (<10 g/dL) | An increase in Hb ≥2 g/dL that is maintained for ≥12 weeks | Same as IWG-MRT-ECNM with allowance of CRh **^a^** |
| Anemia (transfusion-dependent) | Transfusion of ≥6 units PRBCs in the 12 weeks before the start of treatment and Most recent transfusion occurring during the 4 weeks before the start of treatment and Transfusions administered for Hb ≤8.5 g/dL and Reason for transfusions is not bleeding, hemolysis, or therapy-related | Transfusion independence for ≥12 weeks and maintenance of Hgb ≥8.5 g/dL at the end of the 12-week period of response duration | Same as IWG-MRT-ECNM with allowance of CRh **^a^** |
| Thrombocytopenia (transfusion-independent) | ≥Grade 2 thrombocytopenia (<75 × 10^9^/L) | ≥100% increase and an absolute increase ≥50 × 10^9^/L and no need for platelet transfusion for ≥12  weeks | Same as IWG-MRT-ECNM with allowance of CRh **^a^** |
| Thrombocytopenia  (transfusion-  dependent) | Transfusion of ≥6 units of apheresed platelets during 12 weeks preceding treatment and  ≥2 units transfused during 4 weeks preceding treatment and Transfusions administered for platelet count <20 × 109/L | Transfusion independence for  ≥12 weeks and maintenance  of platelet count ≥20 × 109/L | Same as IWG-MRT-ECNM  with allowance of CRh **^a^** |
| **^a^** CRh (CR with partial hematologic recovery) requires the following minimum levels for peripheral blood counts: absolute neutrophil count ≥0.5 × 10^9^/L with normal differential (absence of neoplastic mast cells and blasts < 1%) and platelet count ≥50 × 10^9^/L and hemoglobin ≥8.0 g/dL. Grade is based on the Common Terminology Criteria for Adverse Events, Version 4.03.  Abbreviations: 3D MRI, 3-dimensional magnetic resonance imaging; ALT, alanine aminotransferase; ANC, absolute neutrophil count; ALP, alkaline phosphatase; AST, aspartate aminotransferase; CT, computed tomography; Hgb, hemoglobin; IWG-MRT-ECNM, International Working Group-Myeloproliferative Neoplasms Research and Treatment; LFT, liver function test; MC, mast cell; MRI, magnetic resonance imaging; N/A, not applicable; PBRC, packed red blood cell; ULN, upper limit of normal. | | | |

| **Table S4: Pure Pathologic Response response criteria.^24^** | |
| --- | --- |
| **Response category** | **Definition** |
| Complete remission with full (CR) or  partial (CRh) hematologic recovery **^a^** | Bone marrow mast cell aggregates eliminated and serum tryptase <20 ng/mL |
| Molecular complete remission  (molecular CR/molecular CRh) | KIT D816V mutant allele fraction falls below limit of detection by sensitive assay **^b^** |
| Partial remission (PR) | ≥50% reduction in bone marrow mast cells and serum tryptase level |
| Stable disease (SD) | Not in a CR, PR, or PD |
| Progressive disease (PD) | Transformation to acute myeloid leukemia (AML) or mast cell leukemia (MCL) |
| **^a^** Partial hematologic recovery: ANC > 0.5 × 10^9^/L with normal differential (absence of neoplastic MCs and blasts < 1%) and platelet count >50 × 10^9^/L and Hb level >8.0 g/dL.  **^b^** *KIT* D816V allele-specific polymerase chain reaction or digital droplet assay with sensitivity ~0.1%.  Abbreviations: CR, complete remission; CRh, complete remission with partial hematologic recovery. | |
